# Transcriptomic dynamics changes related to anthocyanin accumulation in the fleshy roots of carmine radish (*Raphanus sativus* L.) characterized using RNA-Seq

**DOI:** 10.7717/peerj.10978

**Published:** 2021-04-07

**Authors:** Xia Song, Jian Gao, Hua Peng

**Affiliations:** 1 Research Center for Tourism Agriculture Development, Sichuan Tourism College, Chengdu, China; 2 School of Advanced Agriculture and Bioengineering, Yangtze Normal University, Fuling, Chongqing, China

**Keywords:** Radish (*Raphanus sativus* L.), Differential expression genes (DEGs), KEGG pathway enrichment, Anthocyanins, Anthocyanin biosynthesis

## Abstract

Carmine radish is famous for containing a natural red pigment (red radish pigment). However, the expression of anthocyanin biosynthesis-related genes during the dynamic development stages of the fleshy roots in carmine radish has not been fully investigated. Here, based on HPLC quantification of anthocyanin levels from our previous study, young fleshy roots of the carmine radish “Hongxin 1” obtained at the dynamic development stages of fleshy roots (seedling stage (SS), initial expansion (IE), full expansion (FE), bolting stage (BS), initial flowering stage (IFS), full bloom stage (FBS) and podding stage (PS)) were used for RNA-Seq. Approximately 126 comodulated DEGs related to anthocyanin biosynthesis (common DEGs in the dynamic growth stages of fleshy roots in carmine radish) were identified, from which most DEGs appeared to be likely to participate in anthocyanin biosynthesis, including two transcription factors, *RsMYB* and *RsRZFP*. In addition, some related proteins, e.g., *RsCHS, RsDFR, RsANS, RsF′3H, RsF3GGT1, Rs3AT1, RsGSTF12, RsUFGT78D2* and *RsUDGT-75C1*, were found as candidate contributors to the regulatory mechanism of anthocyanin synthesis in the fleshy roots of carmine radish. In addition, 11 putative DEGs related to anthocyanin synthesis were evaluated by qRT-PCR via the (2-ΔΔCT) method; the Pearson correlation analysis indicated excellent concordance between the RNA-Seq and qRT-PCR results. Furthermore, GO enrichment analysis showed that “anthocyanin-containing compound biosynthetic process” and “anthocyanin-containing compound metabolic process” were commonly overrepresented in the dynamic growth stages of fleshy roots after the initial expansion stage. Moreover, five significantly enriched pathways were identified among the DEGs in the dynamic growth stages of fleshy roots in carmine radish, namely, flavonoid biosynthesis, flavone and flavonol biosynthesis, diterpenoid biosynthesis, anthocyanin biosynthesis, and benzoxazinoid biosynthesis. In conclusion, these results will expand our understanding of the complex molecular mechanisms of anthocyanin biosynthesis in the fleshy roots of carmine radish and the putative candidate genes involved in this process.

## Introduction

Anthocyanins are water-soluble pigments that produce red to purple colors in nature and belong to the flavonoid group (Khoo et al., 2017). Most research has reported that anthocyanins, which are used as beneficial food additives worldwide, could ameliorate major public health threats (such as cardiovascular disease, inflammation, diabetes and obesity) caused by synthetic food additives (Yousuf et al., 2015; He & Giusti, 2010). Currently, several regulatory genes associated with the anthocyanin biosynthetic pathway have been extensively characterized and found to be conserved among flowering plants (Bajpai et al., 2017). A series of enzymes have been identified to be involved in anthocyanin formation, especially chalcone isomerase (*CHI*), chalcone synthase (*CHS*), dihydroflavonol 4-reductase (DFR), flavanone 3-hydroxylase (*F3H*), and anthocyanidin synthase (*ANS/LDOX*) (Aza-González et al., 2012; Dao & Linthorst, 2011). Fukusaki et al. (2004) reported that flower color would change from blue to white through the knockdown of the *CHS* gene by RNA interference in *Torenia hybrida*. Additionally, in mildly colored pears, the *DFR* and *ANS* genes were identified as important regulators of skin pigmentation (Lepiniec et al., 2006), and various anthocyanidins could be generated from dihydroflavonols using NADPH as a cofactor through the *DFR* and *ANS* genes (Zhang et al., 2011). UDP-glucose: flavonoid 3-O-glucosyltransferase (*UFGT*) was also found to be involved in anthocyanin biosynthesis in grape berries, and the loss of color in white grapes was shown to result from the absence of the *UGFT* gene (Kobayashi et al., 2001). Moreover, several glutathione S-transferases (*GSTs)* were found to be involved in the sequestration of anthocyanins, thereby playing vital roles in flavonoid metabolism in many plants, including Arabidopsis (*Arabidopsis thaliana*), apple (*Malus domestica*) and grape (*Vitis vinifera*) (Soonyoung, Seonae & Yun, 2016; Cutanda-Perez et al., 2009; Li et al., 2011). Additionally, transcription factors (TFs) related to anthocyanin biosynthetic traits act as important regulators in the normal development of an organism and in routine cellular functions (Latchman, 1993; Yusuf et al., 2012). Recently, *MYB-bHLH-WDR* (*MBW*) complexes have been demonstrated to transcriptionally regulate the genes encoding these enzymes through *MYB*, *bHLH* and *WD40* repeats (MBW transcriptional complex) (Xu, Dubos & Lepiniec, 2015). In addition, regulatory genes associated with anthocyanin biosynthesis traits, such as squamosa promoter binding protein-like (*SPL*) (Gou et al., 2011), jasmonate zim-domain (*JAZ*) (Qi et al., 2011), and *NAC* (Zhou et al., 2015), have also been reported in *Arabidopsis thaliana* and blood-fleshed peach. Currently, key anthocyanin biosynthesis-related genes and some of their functions have been extensively characterized in several plants.

Recently, RNA-Seq technology has developed rapidly, and more anthocyanin biosynthesis-related genes have been documented in various fruit crops, including blueberry (Li et al., 2012), blood orange (Crifò, Petrone & Recupero, 2011) and grape (Azuma, 2017). Biosynthesis-related genes in carmine radish (*Raphanus sativus* L.) fleshy roots were identified based on comparative RNA-Seq technology in our previous study (Gao et al., 2020). Specifically, several major anthocyanin biosynthesis-related genes (ABRGs) involved in the regulation of anthocyanin biosynthesis were identified, such as the transcripts of *RsDFR1*, *RsDFR2* and *RsFLS* (Gao et al., 2019a). However, the expression of anthocyanin biosynthesis-related genes related to anthocyanin accumulation in carmine radish (*Raphanus sativus* L.) fleshy roots have not been fully investigated.

## Methods

### Plant material and experimental design

Based on the quantification of anthocyanin levels of carmine radish “Hongxin 1” using HPLC analysis in our previous study (Gao et al., 2019b) (Fig. S1), young fleshy roots obtained from the development stage of carmine radish “Hongxin 1” were used for RNA-Seq in this study. The carmine radish “Hongxin 1” was selected and kept in a greenhouse at the experimental base of the Yangtze Normal University experiment in 2019. First, seeds of “Hongxin 1” were sown in sterile soil under normal growth conditions (23 °C, 16 h light/8 h dark) for 2 weeks. Subsequently, vernalization treatment was conducted, with the 2-week-old plants transferred to and maintained in a cold room (5 ± 1 °C, 12 h light/12 h dark) for 15 days. After vernalization treatment, the plants were grown in a growth room under normal growth conditions (23 °C, 16 h light/8 h dark). Finally, fleshy roots obtained from each stage of development (seedling stage (SS), initial expansion (IE), full expansion (FE), bolting stage (BS), initial flowering stage (IFS); full-bloom stage (FBS) and podding stage (PS)) of carmine radish “Hongxin 1” were collected for RNA-Seq, with three independent biological replicates for each stage and two technical replicates. All harvested tissue was immediately frozen in liquid nitrogen and stored at −80 °C for RNA-Seq analysis.

### Sample preparation and library construction

Library construction was conducted with NEBNext Ultra RNA Library Prep Kits for Illumina (NEB, Ipswich, MA, USA), as follows: The mRNA was isolated with oligo(dT) magnetic beads from approximately 5 µg of total RNA and subsequently converted into short fragments by fragmentation buffer. Then, short fragments were converted into first-strand cDNAs and used as templates for second-strand synthesis with random hexamers. PCR amplification was conducted using the desired purified synthesized cDNA fragments (QiaQuick PCR kit). Ultimately, 200 bp paired-end reads were generated from the prepared library with 2 replicates using Illumina HiSeqTM 2000.

### Read processing and differentially expressed gene (DEG) identification

Using Trimmomatic software, clean reads were obtained by filtering out low-quality reads (base quality ≤10) and adaptor-only reads and then trimming the remaining reads. After that, the small subunit (SSU) and large subunit (LSU) rRNA sequences were downloaded from the Silva database (Christian et al., 2013) and used for high-quality read alignment using BWA software (Li & Richard, 2009), and the mapped rRNA reads were removed by a homemade Perl script. Subsequently, we used Trinity software to de novo assemble the remaining clean reads into transcripts. In addition, BLASTx searches of the Swiss-Prot protein databases and nonredundant protein (Nr) database of the National Center for Biotechnology Information (NCBI) were used to annotate all unigenes to obtain the assembled sequences. The parameters were set as follows: *E*-value < 1e−10, identity > 70%, query coverage ≥ 80%, and default values for other parameters. After gene annotation, the Gene Ontology (GO) database (http://www.geneontology.org/) and Kyoto Encyclopedia of Genes and Genomes (KEGG) database (http://www.kegg.jp/) were used to annotate the functions of assembled unigenes. To assess the abundances of assembled transcripts, we first mapped the clean reads obtained from different fleshy root libraries using Bowtie2 on the de novo-assembled transcriptome. Then, quantification of the de novo assembly transcript was assessed with RSEM, and only transcripts with FPKM ≥ 1 were considered significantly expressed transcripts (Dewey & Li, 2011). Finally, differentially expressed genes (DEGs) with a corrected *P*-value < 0.05 between each set of compared samples were identified through screening by noiseqbio (Tarazona et al., 2012). Furthermore, DEGs related to anthocyanin biosynthesis in carmine radish were analyzed and plotted using neighbor-joining clustering with a homemade R script.

### GO functional annotation and KEGG pathway analysis of comodulated differentially expressed genes (DEGs) in the growth stages of carmine radish

Co-modulated DEGs (DEGs common to all the dynamic growth stages of fleshy roots in carmine radish) were identified based on Venn diagram analysis. Subsequently, we conducted GO annotation of comodulated DEGs through the AgriGO website (http://systemsbiology.cau.edu.cn/agriGOv2/) and KEGG pathway enrichment analysis using KOBAS software (Xie et al., 2011). Their respective graphs were constructed using a homemade R scripts.

### Validation of candidate DEGs involved in the growth stages of carmine radish using real-time qRT-PCR

To validate the results of the RNA-Seq analysis, 11 DEGs with substantial alterations related to the growth stages of carmine radish were chosen for validation by qRT-PCR. The primers used for qRT-PCR experiments were designed by Real-time PCR (TaqMan) Primer and Probes Design Tool (https://www.genscript.com/tools/real-time-pcr-taqman-primer-design-tool), and the radish *ACTIN* gene was used as a reference gene (Table S1). Following the standard protocol for the ABI 7500 system, the amplification of the candidate genes was performed using qRT-PCR in triplicate as described by Jian et al. (2015). The fold change in the expression levels of target genes was calculated using the relative quantitative method (2^−ΔΔCT^) (Schefe et al., 2006), and variance analysis was conducted to evaluate differences in the relative expression levels of the genes between different samples, followed by multiple comparisons using Duncan’s least significant range (LSR) tests with SPSS statistical software. Different letters indicate significant differences at the *p* = 0.05 level. Of those, a, b, c, d, e, f, or g indicates a significant difference from a column with no superscript letter in common (*P* < 0.05); columns marked with the same letter are not significantly different at the 5% level by Duncan’s multiple range test.

## Results

### DEGs related to the dynamic growth stages of fleshy roots in carmine radish

Here, transcriptome analysis of anthocyanin biosynthesis in the carmine radish “Hongxin 1” was conducted followed by HPLC analysis, and young fleshy roots of “Hongxin 1” at different developmental stages were selected for RNA-Seq. The cDNAs obtained from fleshy roots at seven growth phases (SS, IE, FE, BS, IFS, FBS and PS) were sequenced using Illumina sequencing technology. To harvest clean reads for further assembly, we filtered out low-quality reads (base quality ≤10) and adaptor-only reads and then trimmed the sequences to obtain high-quality reads. Subsequently, we aligned the high-quality reads to the small subunit (SSU) and large subunit (LSU) rRNA sequences and removed rRNA reads by a homemade Perl script. After removing rRNA sequences, the average percentage of clean read counts was 70% of raw tags in each library (Table 1).

**Table 1:** Summary of raw reads for 14 samples (two replicates) of fleshy roots of seven growth phases.

Sample	Total_Read_Count	rRNA_Read_Count	Clean_Read_Count	Clean_Rate (%)
FBS_root_1	33,990,858	7,871,020	26,119,838	76.84
FBS_root_2	33,990,858	8,386,243	25,604,615	75.33
BS_root_1	33,401,149	7,702,113	25,699,036	76.94
BS_root_2	33,401,149	7,587,241	25,813,908	77.28
IE_root_1	28,574,219	4,463,319	24,110,900	84.38
IE_root_2	28,574,219	4,663,468	23,910,751	83.68
SS_root_1	20,191,382	504,415	19,686,967	97.50
SS_root_2	20,191,382	560,164	19,631,218	97.23
IFS_root_1	24,547,530	5,534,772	19,012,758	77.45
IFS_root_2	24,547,530	5,976,321	18,571,209	75.65
FE_root_1	14,316,434	3,200,500	11,115,934	77.94
FE_root_2	14,316,434	3,092,331	11,224,103	78.40
PS_root_1	20,673,951	6,219,688	14,454,263	70.08
PS_root_2	20,673,951	5,789,807	14,884,144	72.01

**Figure 1:**
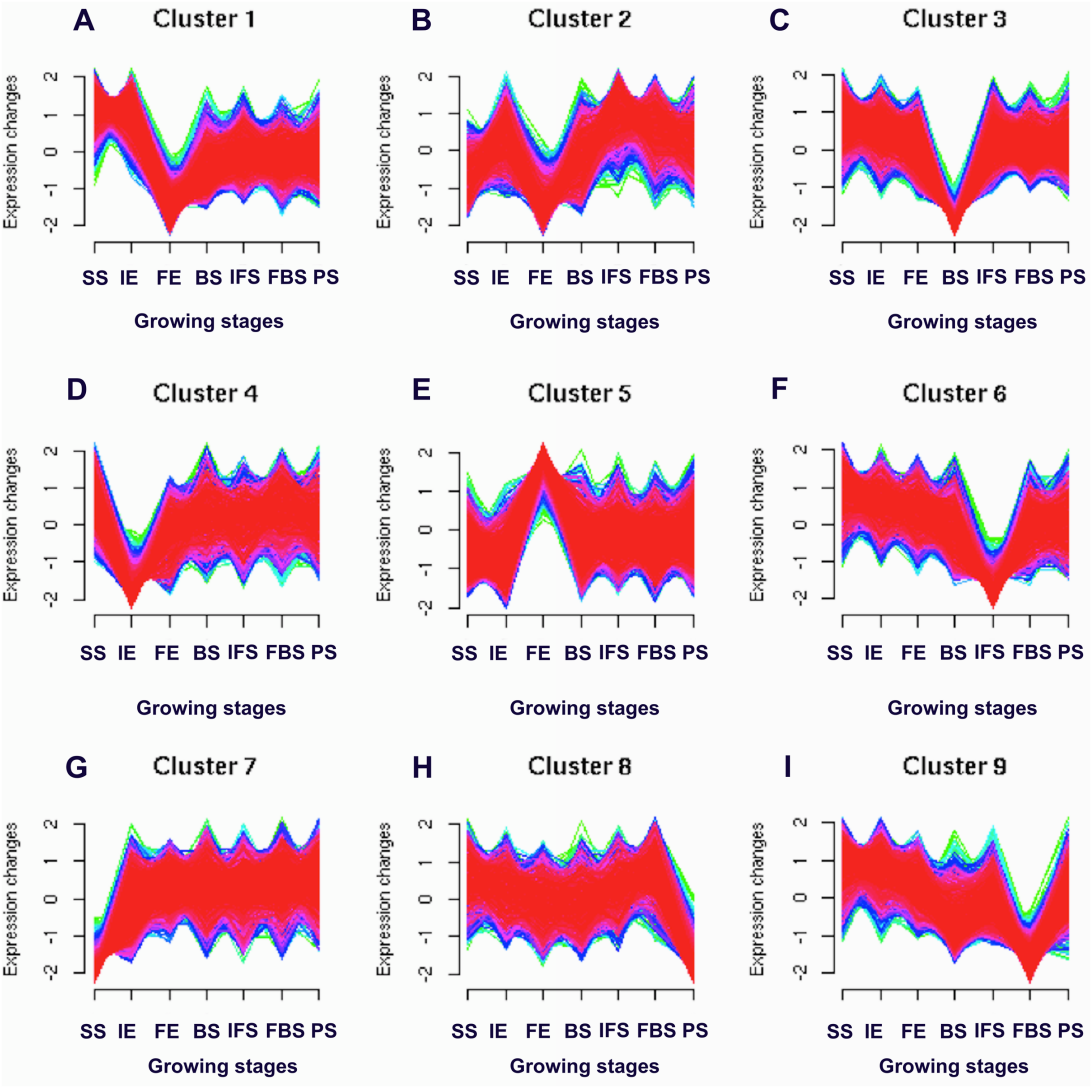
Soft clusters of normalized expression levels for all global expressed genes involved in the dynamics growing stages of fleshy roots in carmine radish. A total of nine clusters (A-I) with the different level of expression changes were identified. Horizontal axis represents growing stages of fleshy roots (“SS_root”, “IE_root”, “FE_root”, “BS_root”, “IFS_root”, “FBS_root” and “PS_root”). The vertical axis represents expression changes.

**Figure 2:**
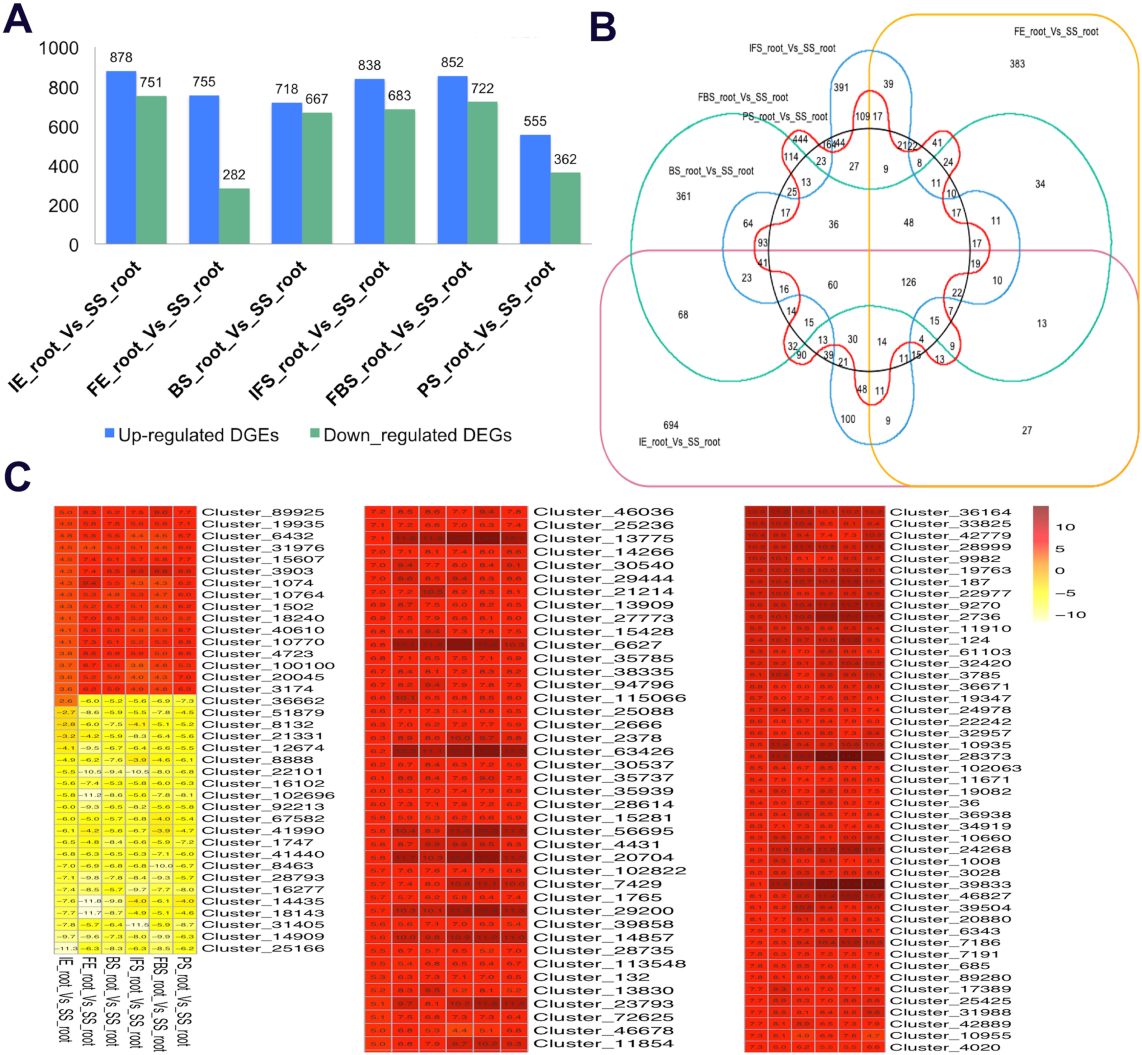
Transcriptional changes of DEGs involved in the dynamics growing stages of fleshy roots in carmine radish. (A) Statistic of differentially expression genes (including up-regulated and down-regulated in each comparison groups) in the dynamics growing stages of fleshy roots (“IE_root”, “FE_root”, “BS_root”, “IFS_root”, “FBS_root” and “PS_root”), compared with “SS_root” group. (B) Venny graph of co-modulated DEGs (Common DEGs in the dynamic growing stages of fleshyroot in carmine radish). (C) Clustering and heat map of common differentially expressed (Co-modulated genes) based on the expression profiles in the dynamics growing stages of fleshy roots (“IE_root”, “FE_root”, “BS_root”, “IFS_root”, “FBS_root” and “PS_root”), compared with “SS_root” group.

To identify the candidate genes related to the dynamic growth stages of fleshy roots in “Hongxin 1” radish, we normalized the expression levels of all the globally expressed genes for further analysis, and the results showed that highly distinct gene expression profiles exist in the dynamic growth stages of fleshy roots (Fig. 1; Table S2). Cluster analysis according to the gene expression dynamics was performed by the k-means method, and differentially expressed genes were divided into 9 clusters associated with distinct expression profiles. More interestingly, we found that the putative candidate genes belonging to Cluster 8 were consistent with the dynamics of the anthocyanin profiles of fleshy roots during the development of carmine radish, while the putative candidate genes categorized into Cluster 9 had the opposite pattern. Furthermore, DEGs related to anthocyanin accumulation in carmine radish (*Raphanus sativus* L.) fleshy roots were identified between other developmental periods (“IE_root”, “FE_root”, “BS_root”, “IFS_root”, “FBS_root” and “PS_root”) and the “SS_root” period. Compared with “SS_root”, 1,629, 1,037, 1,385, 1,521, 1,574 and 917 DEGs were generated in each different periods of development, including both upregulated DEGs (878, 755, 718, 838, 852 and 555 transcripts) and downregulated DEGs (751, 282, 667, 683, 722 and 362 transcripts) (Fig. 2A). Of those, 126 comodulated DEGs were identified based on a Venn diagram (Fig. 2B), and we used a heatmap to display the expression pattern changes of comodulated DEGs (common DEGs involved in the dynamic growth stages of fleshy roots in carmine radish) with different colors (Fig. 2C). Moreover, we found that some comodulated DEGs showed similar expression trends in the dynamic growth stages of fleshy roots, which were again consistent with anthocyanin profiles of the fleshy roots, such as a series of functional enzymes that acted as important regulators in anthocyanin biosynthesis, including dihydroflavonol 4-reductase (*RsDFR*: Cluster_13775), flavonoid 3′-monooxygenase (*RsF3′H*: Cluster_4431), leucoanthocyanidin dioxygenase (*RsLDOX*: Cluster_3903) and chalcone synthase (*RsCHS*: Cluster_39833), as well as some regulatory enzymes, such as anthocyanidin 3-O-glucoside 2′″-O-xylosyltransferase (*Rs3GGT1*: Cluster_9270), coumaroyl-CoA:anthocyanidin 3-O-glucoside-6″-O-coumaroyltransferase 1-like (*Rs3AT1*: Cluster_46827), UDP-glycosyltransferase 75C1-like (*RsUGT75C1*: Cluster_2736) and UDP-glycosyltransferase 78D2-like (*RsUGT78D2*: Cluster_11854). In addition, transport proteins and transcription factors, namely, glutathione S-transferase F12 (*RsGSTF12*: Cluster_24268), MYB transcription factor (*RsMYB2*: Cluster_28373), and zinc finger, RING-type protein (*RsRZEP*: Cluster_7186), were identified (Fig. 2C; Table S3).

### Functional annotation of DEGs related to the dynamic growth stages of fleshy roots in carmine radish

**Figure 3:**
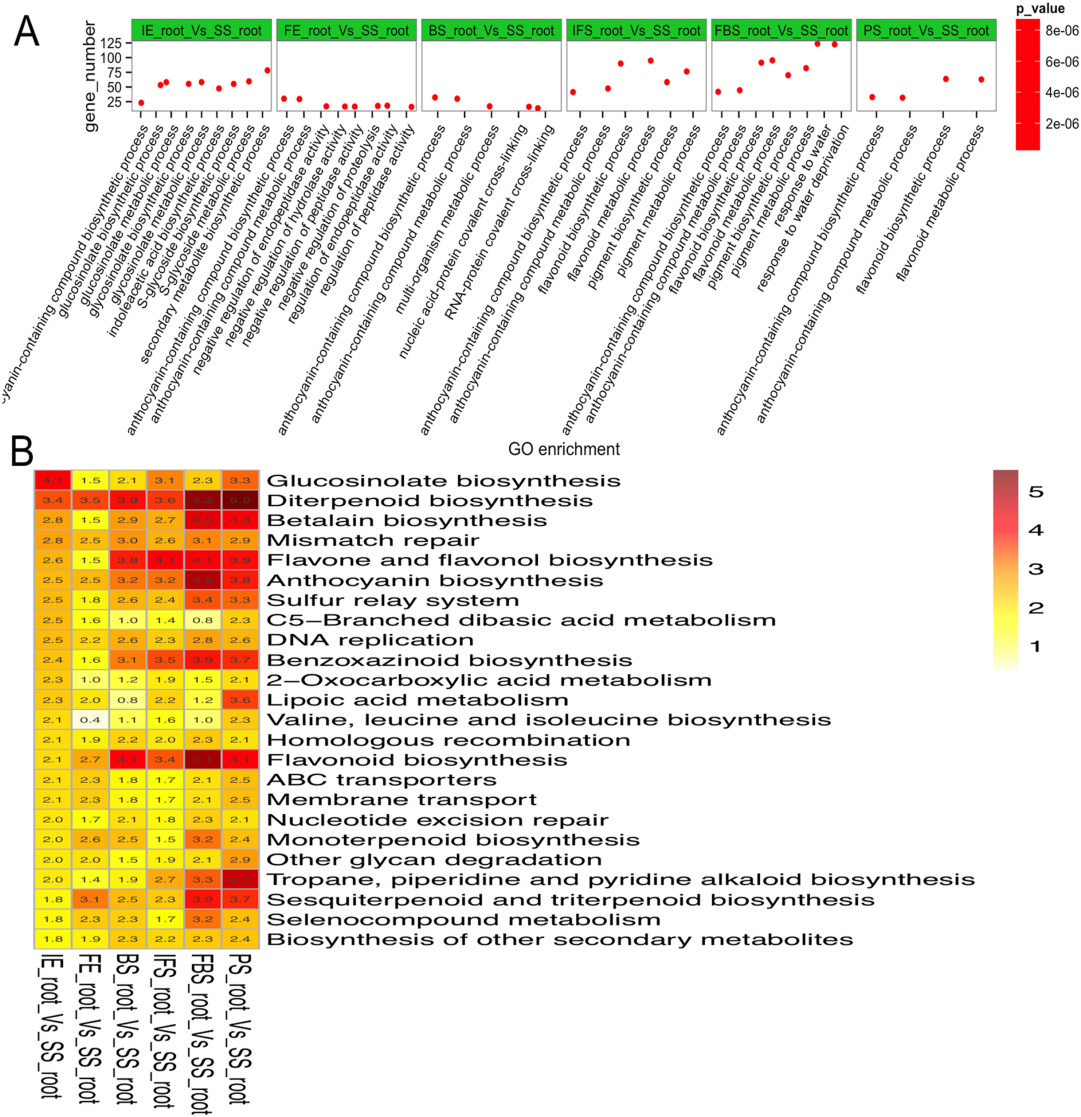
Functional enrichment analysis of differentially expressed genes (DEGs) related to the dynamics growing stages of fleshy roots in carmine radish. (A) Enriched GO terms of DEGs related to the dynamics growing stages of fleshy roots in radish. GO terms are plotted on the ordinate, and the enrichment factor (rich factor) is plotted on the abscissa. The color of points represents the *q*-value, and the size of points represents the number of DEGs mapped to the reference pathway. Legends for the color scale of *q*-values and size-scaling of the number of DEGs are shown to the right of the plot. (B) Pathway enrichment analysis among differentially expressed genes related to anthocyanin synthesis in radish. Enriched KEGG pathway terms divided by the dynamics growing stages (“IE_root”, “FE_root”, “BS_root”, “IFS_root”, “FBS_root” and “PS_root”), compared with “SS_root”. Red color indicates statically overrepresented.

To explore the regulatory mechanisms of DEGs related to the dynamic growth stages of fleshy roots in carmine radish, GO annotation and KEGG pathway enrichment of those putative DEGs were conducted. The results indicated that “anthocyanin-containing compound biosynthetic process (GO:0009718)” and “anthocyanin-containing compound metabolic process (GO:0046283)” were commonly overrepresented in the dynamic growth stages of fleshy roots after the initial expansion (i.e., 40 days after planting), “flavonoid biosynthetic process (GO:0009813)” and “flavonoid metabolic process (GO:0009812)” were overrepresented in IFS, FBS and PS, but “pigment biosynthetic process (GO:0046148)” and “pigment metabolic process (GO:0042440)” were overrepresented in IFS and FBS. Moreover, we found that the GO terms “glucosinolate biosynthetic process (GO:0019758)” and “glucosinolate metabolic process (GO:0019757)” were overrepresented in IE (Fig. 3A; Table S4). KEGG pathway enrichment analysis identified five significantly enriched pathways related to anthocyanin synthesis traits in carmine radish, including flavonoid biosynthesis, flavone and flavonol biosynthesis, diterpenoid biosynthesis, anthocyanin biosynthesis, and benzoxazinoid biosynthesis (Fig. 3B; Table S5).

### Validation of DEGs related to anthocyanin synthesis traits through qRT-PCR

**Figure 4:**
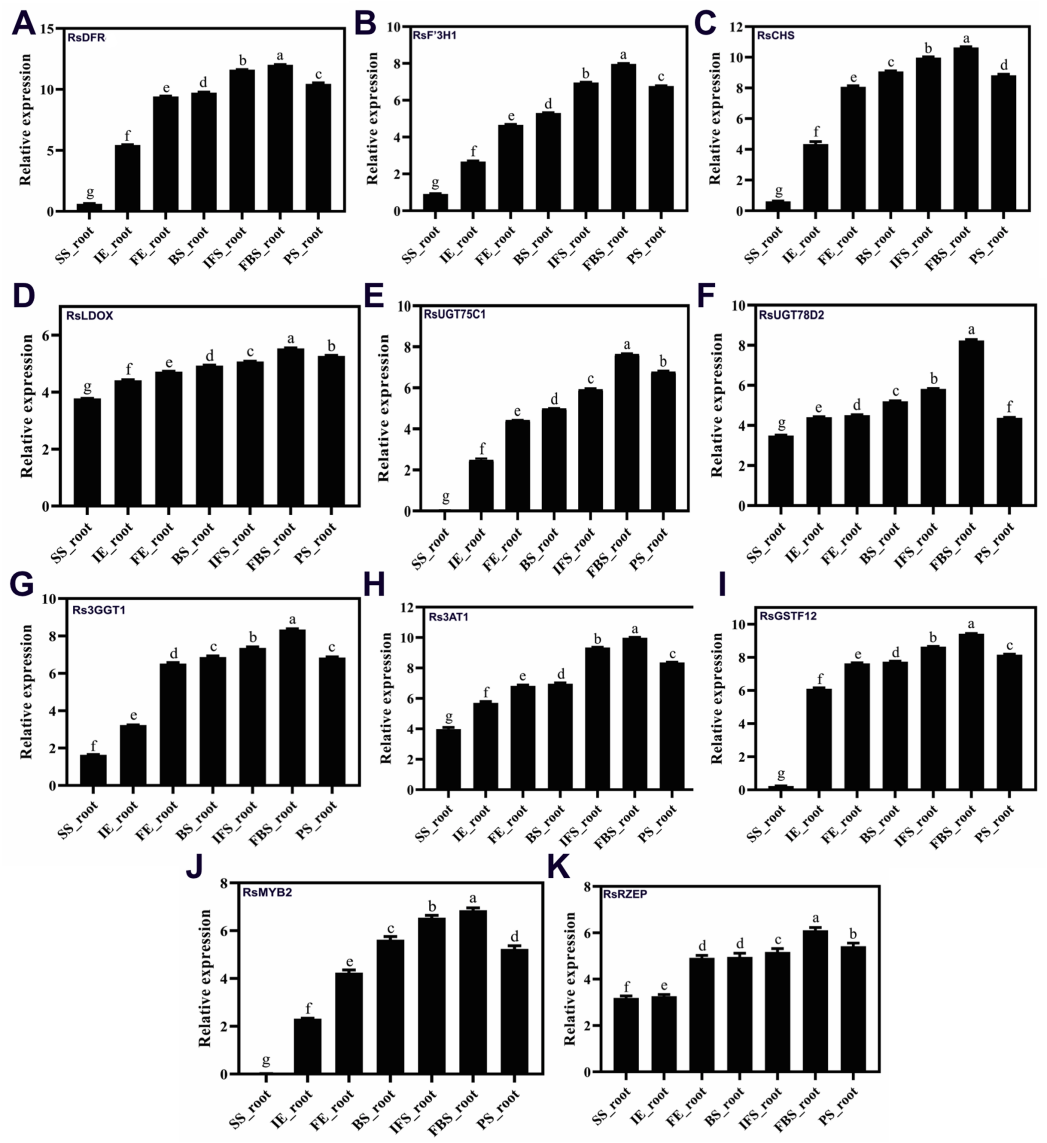
(A–K) Transcriptional analysis of anthocyanin synthesis-related genes (ASRGs) identified in fleshy roots obtained from the dynamics development stage of fleshy roots (seedling stage (SS), initial expansion (IE), full-expansion (FE), bolting stage (BS), initial. flowering stage (IFS); full-bloom stage (FBS) and podding stage (PS)) in carmine radish “Hongxin 1” using qRT-PCR. Relative gene expression levels were normalized against actin transcript levels, and log2 scale for fold change of gene expression in the development stage of fleshy roots comprising of “SS”, “IE”, “FE”, “BS”, “IFS”, “FBS” and “PS” was shown. The standard error calculated from three biological replicates and significant (*P* < 0.05) difference identified by uncorrected Fisher’s LSD test in multiple comparisons after two-way ANOVA are indicated by error bars and stars, respectively.

**Figure 5:**
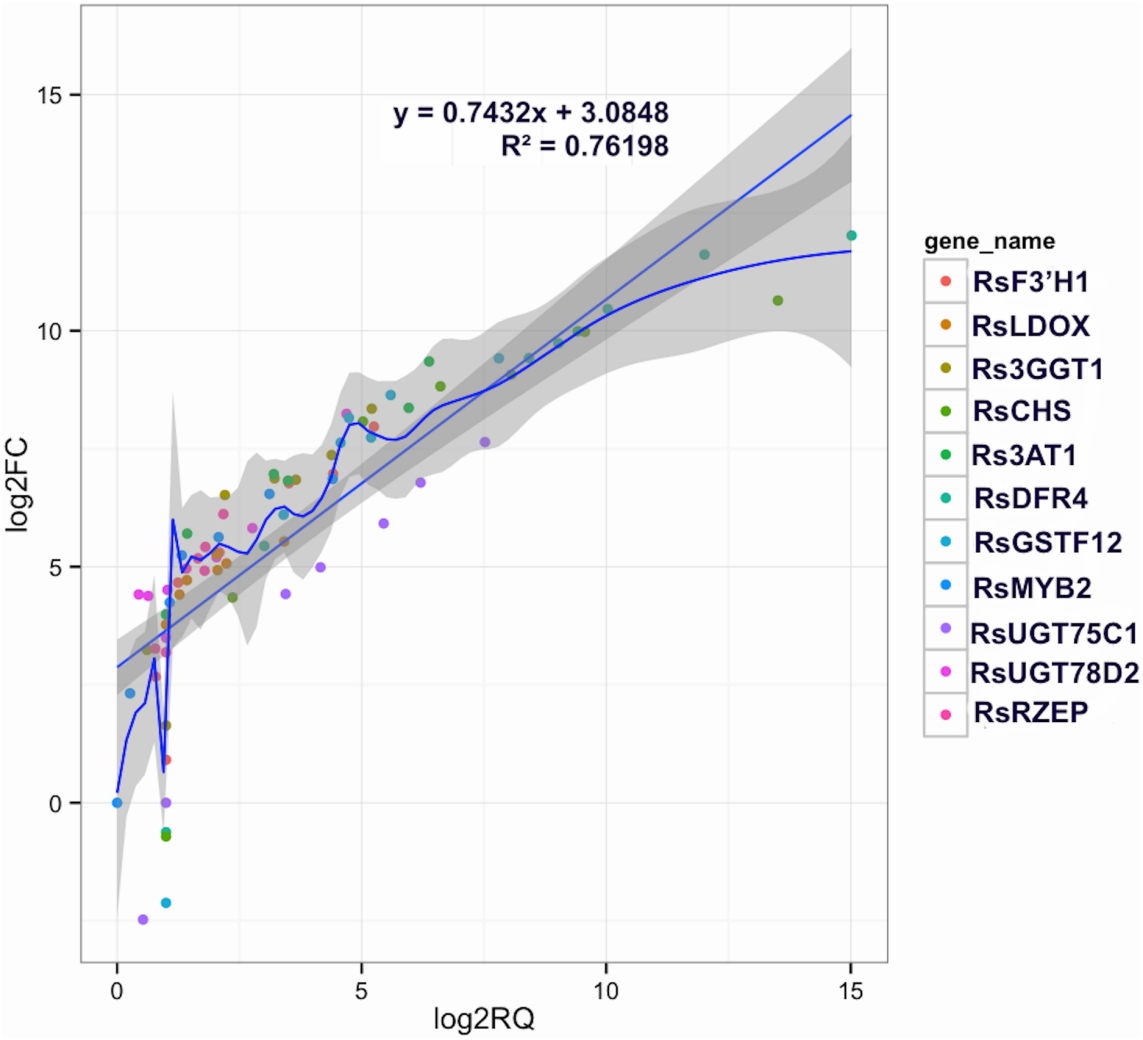
Correlations of transcript levels of candidate DEGs related to anthocyanin synthesis traits in carmine radish between RNA-seq and qPCR data. Validation of candidate Co-modulated DEGs involved in the dynamics growing stages of fleshy roots in carmine radish using qRT-PCR and then correlation between RNA-seq and qPCR data were conducted. Each RNA-seq expression data was plotted against that from quantitative real-time PCR and fit into a linear regression. Both *x*- and *y*-axes were shown in log2 scale and each color represented a different gene.

To confirm the RNA-Seq results, eleven putative DEGs (Cluster_13775, Cluster_4431, Cluster_3903, Cluster_39833, Cluster_9270, Cluster_46827, Cluster_2736, Cluster_11854, Cluster_24268, Cluster_28373 and Cluster_7186) were selected and subjected to qRT-PCR analysis (Fig. 4). The changes in expression of these 11 putative DEGs related to anthocyanin synthesis were measured by log2-fold change, and the Pearson correlation analysis indicated excellent concordance between RNA-Seq and qRT-PCR (R2 = 0.76198) (Table S6; Fig. 5). Here, transcripts of dihydroflavonol 4-reductase (*RsDFR*: Cluster_13775), flavonoid 3′-monooxygenase (*RsF3′H*: Cluster_4431), chalcone synthase (*RsCHS*: Cluster_39833) and leucoanthocyanidin dioxygenase (*RsLDOX*: Cluster_3903) were identified and validated using qRT-PCR. The transcripts of *RsDFR*, *RsF3′H* and *RsCHS*, but not *RsLDOX*, were significantly upregulated in the different growth stages of fleshy roots among other different development periods from seedling stage to full-bloom stage but decreased in the podding stage, showing consistency with the dynamics of anthocyanin profiles of fleshy root in development of carmine radish (Figs. 4A, 4B, 4C and 4D); similar patterns were observed for UDP-glycosyltransferase 75C1-like (*RsUGT75C1*: Cluster_2736) and UDP-glycosyltransferase 78D2-like (*RsUGT78D2*: Cluster_11854), which have been reported to be involved in the methylation of anthocyanidins, resulting in stable compounds. We found that the expression of *RsUGT75C1* was significantly different from that of *RsUGT78D2* during the fleshy root growth development stages compared with the seedling stage (Figs. 4E and 4F). In addition, anthocyanidin 3-O-glucoside 2′″-O-xylosyltransferase (*Rs3GGT1*: Cluster_9270) and coumaroyl-CoA:anthocyanidin 3-O-glucoside-6″-O-coumaroyltransferase 1-like (*Rs3AT2*: Cluster_9270) might act as key regulatory enzymes for the formation of anthocyanins in carmine radish. We found that *Rs3GGT1* was changed significantly compared with *Rs3AT2* (Figs. 4G and 4H). Moreover, transport proteins and transcription factors such as glutathione S-transferase F12 (*RsGSTF12*: Cluster_24268), MYB transcription factor (*RsMYB2*: Cluster_28373), and zinc finger, RING-type protein (*RsRZEP*: Cluster_7186) were also identified. The transcripts of *RsGSTF12* and *RsRZEP* were not significantly differentially expressed in the other growth stages of fleshy roots compared with the seedling stage (Figs. 4I and 4K), but we found that the transcript of *RsMYB2* (Fig. 4J) was significantly upregulated in the fleshy root growth stage and decreased in podding stage compared with other development periods from seedling stage to full-bloom stage. This pattern was consistent with the dynamics of anthocyanidin profiles across the fleshy root development stages in the carmine radish.

## Discussion

Anthocyanins are major color pigments and play diverse physiological roles in plants. Currently, more than 550 different anthocyanins have been isolated from diverse plants and categorized by the extent of hydroxylation in the flavonoid B ring (Ferreira, Slade & Marais, 2005). Studies have demonstrated that anthocyanin compounds are synthesized through the methylation, glycosylation and acylation of the basic flavonol structure. Several structural genes and enzymes related to anthocyanin and flavonoid biosynthesis have been reported in most plant species. Previous studies have reported that anthocyanins are first formed from phenylalanine by a series of phenylpropanoid metabolism enzymes, such as chalcone synthase (*CHS*), the key enzyme in the first step of flavonoid synthesis, followed by chalcone isomerase (*CHI*), which leads to the formation of flavanones by closing the C-ring (Grotewold, 2006). After that, proanthocyanidins important for the formation of anthocyanidins were produced through anthocyanidin synthase (*ANS*) and dihydroflavonol 4-reductase (*DFR*), and subsequently, anthocyanins were produced through the action of UDP-flavonoid glycosyltransferases (*UFGTs*) (Xie, 2003). In our previous study, de novo transcriptome sequencing of radish (*Raphanus sativus* L.) fleshy roots showed that the majority of anthocyanin biosynthesis-related genes (ABRGs) are involved in the regulation of anthocyanin biosynthesis. Of those, *RsPAL2*, *RsCHS-B2*, *RsDFR1*, *RsDFR2*, *RsFLS*, *RsMT3* and *RsUFGT73B2-like* were identified as significantly associated with anthocyanin biosynthesis. *RsDFR1* and *RsDFR2* were most highly enriched in HX-3, and *RsFLS*, *RsDFR1* and *RsDFR2* were enriched in WG-3, but *RsFLS* was downregulated in HX-3 and WG-3. We proposed that *RsDFR1*, *RsDFR2* and *RsFLS* might act as key regulators in the anthocyanin biosynthesis pathway (Gao et al., 2019a). In addition, several differentially expressed genes related to anthocyanin biosynthesis in carmine radish (*Raphanus sativus* L.) fleshy roots were identified using comparative RNA-Seq, including *RsCHS*, *RsCHI*, *RsANS*, *RsMT2-4*, *RsUF3GT*, glutathione S-transferase F12, *RsUFGT78D2-like* and *RsUDGT-75C1-like*, and these genes likely significantly contribute to the regulation of anthocyanin biosynthesis in radish cultivars (Gao et al., 2020). The dihydroflavonol 4-reductase (*RsDFR*: Cluster_13775), flavonoid 3′-monooxygenase (*RsF3’H*: Cluster_4431), leucoanthocyanidin dioxygenase (*RsLDOX*: Cluster_3903) and chalcone synthase (*RsCHS*: Cluster_39833) genes were identified and validated using qRT-PCR. These genes were significantly upregulated during the dynamic growth stages of fleshy roots relative to the other development periods from the seedling stage to the full-bloom stage but decreased in the podding stage, consistent with the dynamics of the anthocyanidin profile. In addition, the expression of UFGT genes has also been identified as an important regulatory mechanism involved in the anthocyanin biosynthetic pathway (Cutanda-Perez et al., 2009; Griesser et al., 2008) as UGFTs attach sugar moieties to the anthocyanin aglycone to stabilize anthocyanidin. In this study, UDP-glycosyltransferase 75C1-like (*RsUGT75C1*: Cluster_2736) and UDP-glycosyltransferase 78D2-like (*RsUGT78D2*: Cluster_11854) were significantly upregulated in different fleshy root types with the development of dynamic growth stages of fleshy roots from the seedling stage to the full-bloom stage but decreased in the podding stage. Further modifications involving glycosylation, acylation or methylation can be made; for example, anthocyanin is formed through a reaction catalyzed by a cyanidin 3-O-glycosyltransferase (Ni et al., 2018). Here, anthocyanidin 3-O-glucoside 2′″-O-xylosyltransferase (*Rs3GGT1*: Cluster_9270), coumaroyl-CoA:anthocyanidin 3-O-glucoside-6″-O-coumaroyltransferase 1-like (*Rs3AT2*: cluster_9270) were expressed as key regulatory enzymes for the formation of anthocyanins in carmine radish. In addition, transport proteins and transcription factors comprising glutathione S-transferase F12 (*RsGSTF12*: Cluster_24268), MYB transcription factor (*RsMYB2*: Cluster_28373), and zinc finger, RING-type protein (*RsRZEP*: Cluster_7186) were identified. Based on genetic and biochemical evidence, several GSTs were found to be involved in anthocyanin transport (Zhao & Dixon, 2010). Bz2 (encoded by the GST gene) was first found to act as an important regulator in the vacuolar transfer of anthocyanins in *Zea mays* (Marrs, Alfenito & Lloyd, 1995). In addition, anthocyanin accumulation and pigment mislocalization were found to be induced by mutations in GST-encoding genes in Arabidopsis (Kitamura, Shikazono & Tanaka, 2010). Here, we found that *RsGSTF12* was significantly upregulated in different stages of fleshy root development. Moreover, MYB acts as a central regulator for determining the variation in anthocyanin production (Espley et al., 2009). A previous study demonstrated that the transcription of both early (*CHS*, *CHI*, *F3′H* and *FLS*) and late (*DFR*, *ANS* and *ANR*) flavonoid biosynthesis genes in the anthocyanin pathway was directly activated by R2R3-type MYB proteins and the MYB-bHLH-WD40 complex (Xu, Dubos & Lepiniec, 2015). Here, *RsMYB2* was found to be significantly over-expressed and remarkably positively correlated with red pigment content in the dynamic development of fleshy roots in carmine radish. We therefore infer that *RsMYB2* transcription factors might specifically activate flavonoid biosynthesis genes (*RsCHS*, *RsF3′H*, *RsDFR*) to regulate anthocyanin biosynthesis in carmine radish. However, the molecular mechanism of *RsCHS*, *RsF3′H*, and *RsDFR* regulation by *RsMYB2* needs further study.

## Conclusion

RNA-Seq technology was applied to identify the key anthocyanin biosynthesis-related genes involved in the regulation of anthocyanin biosynthesis during the dynamic growth stages of fleshy roots in carmine radish. Of those, two transcription factors, *RsMYB2* and *RsRZFP*, as well as some related functional genes, e.g., *RsCHS, RsDFR, RsLDOX, RsF′3H, RsF3GGT1, Rs3AT1, RsGSTF12, RsUFGT78D2* and *RsUDGT-75C1*, may contribute to the regulatory mechanism of anthocyanin synthesis. In addition, Pearson correlation analysis indicated excellent concordance between RNA-Seq and qRT-PCR experiments. Moreover, qRT-PCR showed that *RsCHS, RsDFR, RsF′3H, RsUDGT-75C1, RsF3GGT1, Rs3AT1, RsMYB2* and *RsRZFP* were significantly upregulated and showed a remarkable positive correlation with the red pigment content in fleshy roots at different stages. We proposed that *RsCHS, RsDFR, RsF′3H, RsUDGT-75C1, RsF3GGT1, Rs3AT1*, and *RsMYB2* might act as key regulators in the anthocyanin biosynthesis pathway.

## Supplemental Information

10.7717/peerj.10978/supp-1 Supplemental Information 1 RNAseq data analysis and qRT-PCR data.

10.7717/peerj.10978/supp-2 Supplemental Information 2 qRT-PCR data for candidate DEGs. qRT-PCR data for 11 selected DEGs related to anthocyanin biosythesis.

10.7717/peerj.10978/supp-3 Supplemental Information 3 Sequence data for all cluster IDs.

10.7717/peerj.10978/supp-4 Supplemental Information 4 Dynamics anthocyanin profiles of fleshy roots in development of carmine radish (‘Hongxin 1’) and their responding diagram of developmental stages of fleshy roots.

10.7717/peerj.10978/supp-5 Supplemental Information 5 KEGG enrichment of all unigene assembled in this study.

10.7717/peerj.10978/supp-6 Supplemental Information 6 Gene annotation and description of all unigenes identified in this study.

10.7717/peerj.10978/supp-7 Supplemental Information 7 Go annotation of all unigenes assembled in this study.

10.7717/peerj.10978/supp-8 Supplemental Information 8 Raw data of pigment content.

## List of Abbreviations

ASRGs: Anthocyanin synthesis-related genes
PAL: Phenylalanine ammonia-lyase
C4H: Cinnamate 4-hydroxylase
4CL: 4-coumarate: CoA ligase
CHS: Chalcone synthase
CHI: Chalcone isomerase
F3H: Flavanone 3-hydroxylase
F35H: Flavonoid 3,5 –hydroxylase
DFR: Dihydroflavonols 4-reductase
ANS: Anthocyanin synthase
UFGT: UDP-glucose Flavonoid 3-glucosyltransferase
FNS: Flavone synthase
FLS: Flavonol synthase
MT: Metallothionein-like protein
SS: seedling stage
IE: initial expansion
FE: full expansion
BS: bolting stage
IFS: initial flowering stage
FBS: full-bloom stage
PS: podding stage
DEGs: differentially expressed genes
KEGG: Kyoto Encyclopedia of Genes and Genomes
FPKM: fragments per kilobase of transcript per million mapped reads
GO: Gene Ontology
NCBI: National Center for Biotechnology Information
BP: biological process
MF: molecular function
CC: cellular component

## Additional Information and Declarations

### Data Availability

The following information was supplied regarding data availability: All the raw read sequences are available at the NCBI Sequence Read Archive: PRJNA565866.
