# Supplementary figures and images for: Transcriptomic dynamics changes related to anthocyanin accumulation in the fleshy roots of carmine radish (Raphanus sativus L.) characterized using RNA-Seq
Source: PeerJ. 2021 Apr 7;9:e10978. doi: 10.7717/peerj.10978 (PMC8035900; doi:10.7717/peerj.10978)

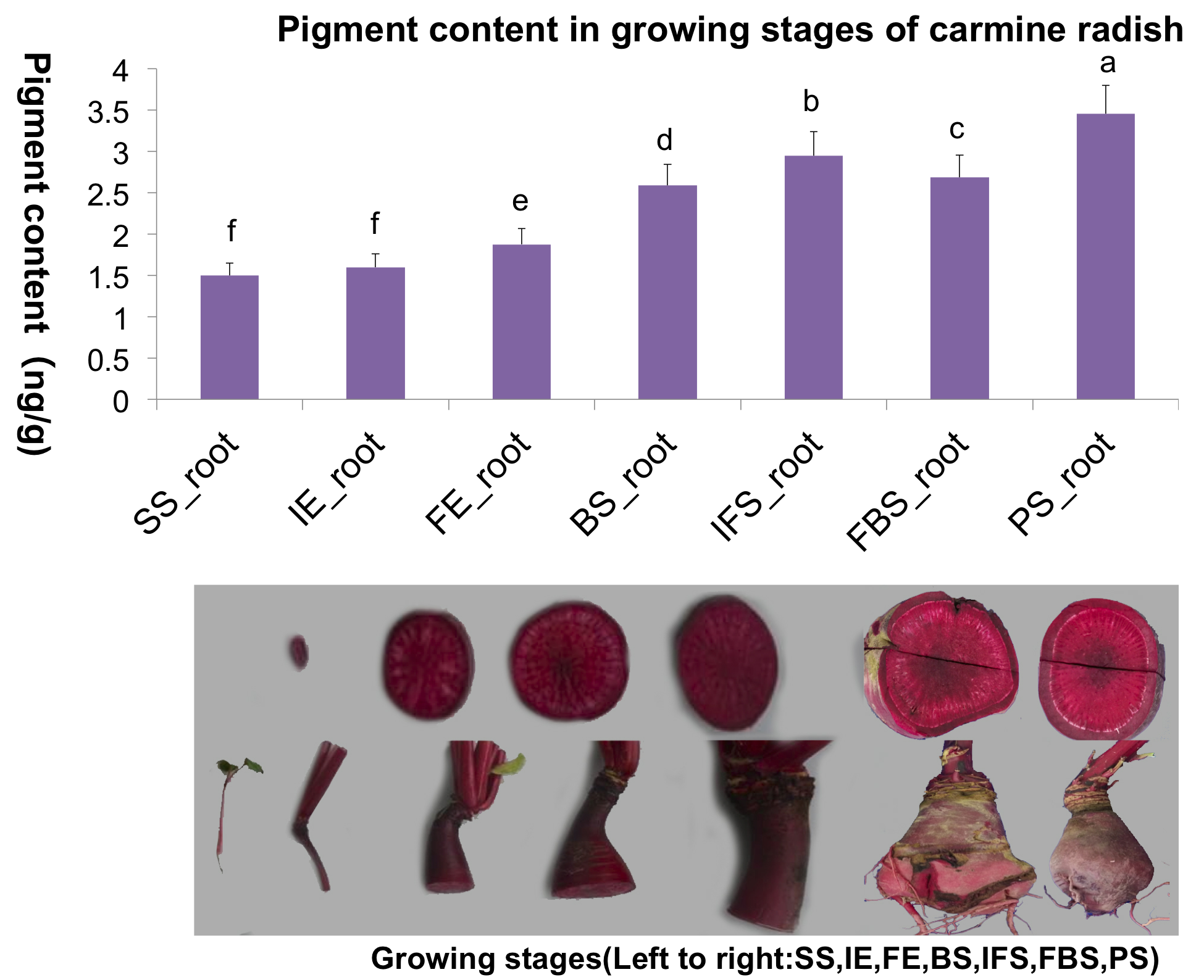

Supplement: Supplemental Information 4 [file peerj-09-10978-s004.png]
